# Classification of motor imagery EEG using deep learning increases performance in inefficient BCI users

**DOI:** 10.1371/journal.pone.0268880

**Published:** 2022-07-22

**Authors:** Navneet Tibrewal, Nikki Leeuwis, Maryam Alimardani

**Affiliations:** 1 Department of Cognitive Science and Artificial Intelligence, Tilburg University, Tilburg, The Netherlands; 2 Research Department, Unravel Research, Utrecht, The Netherlands; Effat University, SAUDI ARABIA

## Abstract

Motor Imagery Brain-Computer Interfaces (MI-BCIs) are AI-driven systems that capture brain activity patterns associated with mental imagination of movement and convert them into commands for external devices. Traditionally, MI-BCIs operate on Machine Learning (ML) algorithms, which require extensive signal processing and feature engineering to extract changes in sensorimotor rhythms (SMR). In recent years, Deep Learning (DL) models have gained popularity for EEG classification as they provide a solution for automatic extraction of spatio-temporal features in the signals. However, past BCI studies that employed DL models, only attempted them with a small group of participants, without investigating the effectiveness of this approach for different user groups such as inefficient users. BCI inefficiency is a known and unsolved problem within BCI literature, generally defined as the inability of the user to produce the desired SMR patterns for the BCI classifier. In this study, we evaluated the effectiveness of DL models in capturing MI features particularly in the inefficient users. EEG signals from 54 subjects who performed a MI task of left- or right-hand grasp were recorded to compare the performance of two classification approaches; a ML approach vs. a DL approach. In the ML approach, Common Spatial Patterns (CSP) was used for feature extraction and then Linear Discriminant Analysis (LDA) model was employed for binary classification of the MI task. In the DL approach, a Convolutional Neural Network (CNN) model was constructed on the raw EEG signals. Additionally, subjects were divided into high vs. low performers based on their online BCI accuracy and the difference between the two classifiers’ performance was compared between groups. Our results showed that the CNN model improved the classification accuracy for all subjects within the range of 2.37 to 28.28%, but more importantly, this improvement was significantly larger for low performers. Our findings show promise for employment of DL models on raw EEG signals in future MI-BCI systems, particularly for BCI inefficient users who are unable to produce desired sensorimotor patterns for conventional ML approaches.

## 1 Introduction

Motor Imagery (MI) is a dynamic experience where the user contemplates mental imagination of motor movement without activation of any muscle or peripheral nerve. A Motor Imagery Brain-Computer Interface (MI-BCI) serves as a system that converts brain signals generated during such imagination into an actionable sequence [1–4].

MI-BCI systems mainly utilize electroencephalogram (EEG) for measurement of brain activity [5]. EEG provides high temporal resolution, can be portable, is relatively low cost and represents synchronous electrical signals produced by the brain [5]. However, the recorded EEG signals are non-stationary and suffer from a low signal-to-noise ratio (SNR) and poor spatial resolution. Therefore, in order to employ them in a BCI system, it is necessary to apply advanced signal processing techniques to clean the data from artefacts and extract relevant spatial, temporal and frequency information from the signals for the classification problem [6].

Traditionally, MI-BCIs operate on machine learning (ML) algorithms in which spatial features associated with movement imagination are recognized. The imagining of a left or right body movement is accompanied by a lateralization of event-related (de)synchronization (ERD/ERS) in the mu (7–13 Hz) and beta (13–30 Hz) frequency bands of EEG signals [7–10]. This brain activity feature is usually picked up by the Common Spatial Pattern (CSP) algorithm [11] and serves as an input to the ML algorithm classifying the imagined body movements. Therefore, the system relies on the user to consciously modulate their brain activity such that the lateralization can be detected.

However, it is known that fifteen to thirty percent of users cannot accomplish distinctive brain waves such that the classifier reaches accuracy above 70%. This is called ‘*BCI illiteracy*’ [12] or ‘*BCI inefficiency*’ [13], where a user is considered unable to control a BCI, even after extensive training. While multiple studies have focused on identifying the inefficient users early on in research or adapting the BCI training to them [e.g., 14–16], the issue of BCI inefficiency might be argued more nuanced; as successful BCI control depends on a synergy between man and machine, and therefore enhancements on both sides are needed to reach efficient control [13].

To that end, deep Learning (DL) classifiers present a promising alternative to address the complexity of EEG signals underlying MI task and its variability among users, as they can work with raw data and directly learn features and capture structure of a large dataset without any feature engineering or selection processes [17–20]. Thus, the issue of information loss while generating and selecting features is avoided when DL classifiers are used [21]. Additionally, they can be used to stabilize the learning process by overcoming the issue of noise and outliers in the data [22]. DL generates high-level abstract features from low-level features by identifying distributed patterns in the acquired data. Hence, DL models hold the potential of handling complex and non-linear high dimensional data [10].

Past research has already established the effectiveness of the DL approach, especially Convolutional Neural Network (CNN), in classification of MI-EEG [23–32]. The advantages of CNN model include handling raw data without any feature engineering process, facilitating end-to-end learning and requiring lesser parameters than other deep neural networks [17, 33]. CNN works well with large datasets and can exploit the hierarchical structure in natural signals [34]. Moreover, CNN has good regularization and degree of translation invariance properties along with the ability to capture spatial and temporal dependencies of EEG signals [35].

However, while CNN has generally proven to be effective in EEG classification, its contribution to improve BCI performance for inefficient users remains unexplored. In a recent study, Stieger et al. [28] showed a negative correlation between online (ML-based) performance and improvement of accuracy with CNN, which suggests that BCI inefficient users may benefit from applying a DL classifier, even more than high aptitude users. They further showed that the low performing users in the online classification did not necessarily produce the expected SMR activity during the MI process, but instead produced differentiating activity over brain regions outside the motor cortex such as occipital and frontal gamma power, which could not be recognized by the feature extraction algorithm. Therefore, DL models might be particularly beneficial to inefficient users by identifying EEG pattern changes that are not recognizable by traditional ML approaches.

This study aims to elucidate the effectiveness of DL over ML in classification of MI EEG signals particularly for inefficient users, by comparing the two classification approaches in a large group of 54 subjects including both high and low performing users. For every subject, a CNN model (DL approach) was trained and its performance was compared with the conventional CSP+LDA model (ML approach), which is widely used in binary MI-BCI classification [36–39].

**Fig 1:**
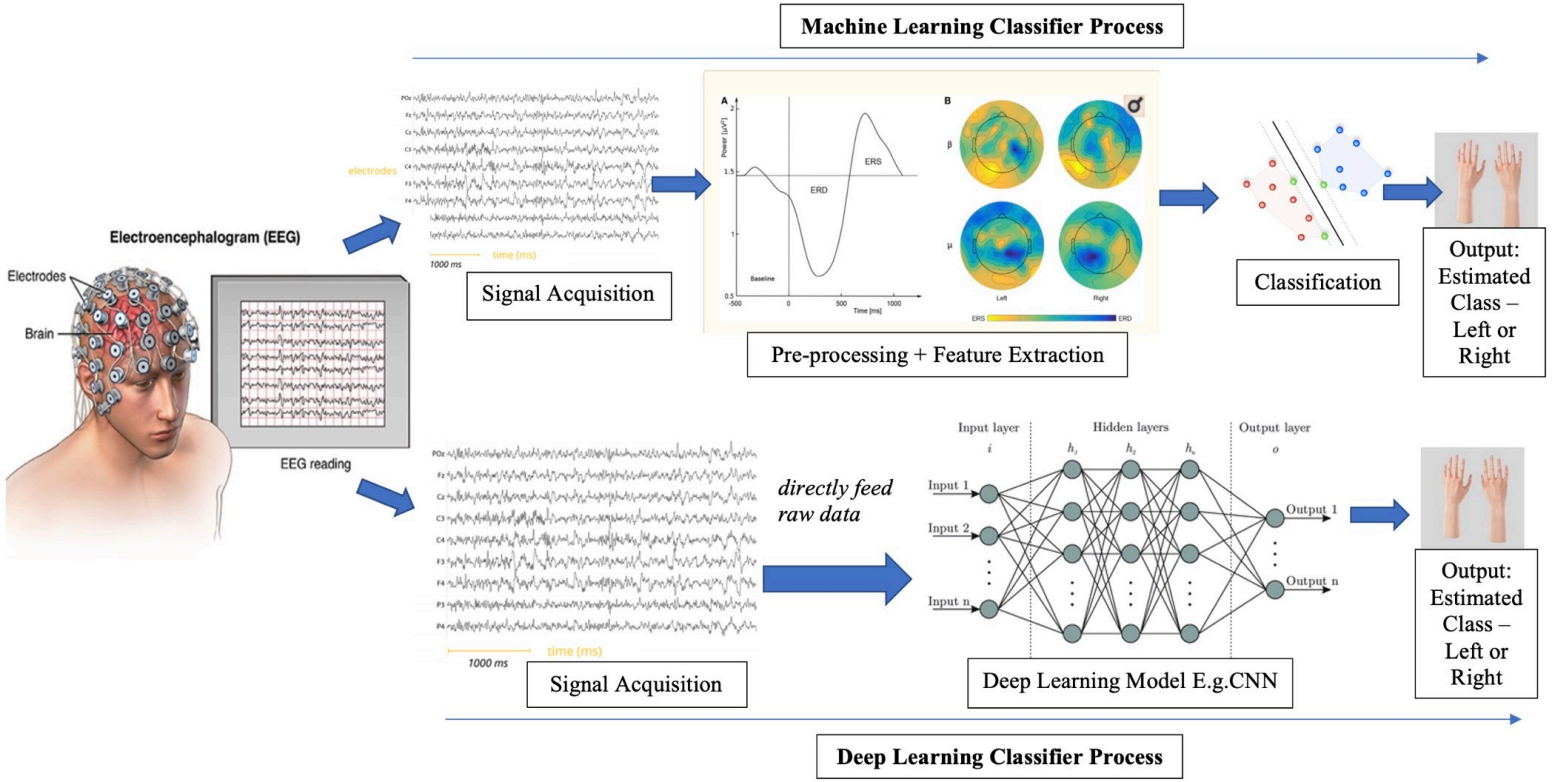
An overview of MI-BCI classification using machine learning vs. deep learning approaches. In ML approach, EEG signals are first pre-processed and relevant features are extracted before applying a classifier. In DL approach, raw signals are directly fed into the model.

Fig 1 shows sequential steps that were taken in each approach to construct a MI-BCI classifier and obtain classification performances. The ‘*Signal Acquisition*’ step was carried out through EEG to monitor the brain signals arising from the mental image of the movement by the user. The complexity of the ML approach arises with the steps involved in ‘*Pre-processing*’ and ‘*Feature Extraction*,’ whereas in the DL approach, raw data can directly be fed into the model. Hence, by applying both approaches to the data from 54 subjects, this study intends to answer the following research questions:

RQ1: *Can a CNN classifier trained with raw EEG signals achieve a higher performance than a machine learning model that runs on processed EEG features for classification of a two-class Motor Imagery task*?
RQ2: *Can a CNN classifier improve BCI performance for inefficient BCI users more than efficient users*?

By evaluating the effectiveness of the deep learning approach for each user group, our study aims to provide evidence that DL models can serve as a promising tool in identifying EEG pattern changes in inefficient users and hence enhancing overall BCI usability.

## 2 Methods

In order to compare conventional ML models with a DL approach, EEG signals were collected from 57 subjects while they performed the MI task using an existing BCI system. Thereon, the recorded EEG signals were used to train a CNN and CSP+LDA model to conduct an offline classification of two-class MI task. Additionally, online accuracies obtained from the BCI system were further employed as the real-time performance of the classifier to determine whether the user belonged to the low performers or high performers group. The following section gives a description of the data collection procedure and details of the classification models.

### 2.1 Experiment

#### 2.1.1 Participants

In this experiment, 57 subjects participated (21 male, 36 female, *M*_*age*_ = 20.71, *SD*_*age*_ = 3.52) [15, 40]. All of them were right-handed and novice to BCI and the MI task. The Research Ethics Committee of Tilburg School of Humanities and Digital Sciences approved the study (REDC #20201003). All subjects received explanation regarding experiment procedure and signed a consent form before the experiment.

#### 2.1.2 EEG acquisition

Sixteen electrodes recorded EEG signals from the sensorimotor area according to the 10–20 international system (F3, Fz, F4, FC1, FC5, FC2, FC6, C3, Cz, C4, CP1, CP5, CP2, CP6, T7, T8). The right earlobe was used as a reference electrode and a ground electrode was set on AFz. Conductive gel was applied to keep the impedance of the electrodes below 50 kOhm. Subjects were instructed to sit calmly and avoid movements and excessive blinking. The signals were amplified by a g.Nautilus amplifier (g.tec Medical Engineering, Austria). The data was sampled at 250 samples/second. The noise during EEG recording was reduced by applying a 48–52 Hz notch filter and 0.5–30 Hz bandpass filter.

#### 2.1.3 Motor imagery task

Participants performed the MI task in four runs, each consisting of 20 right- and 20 left-hand trials. The first run was a non-feedback run, followed by three runs in which the subjects received feedback in form of a feedback bar on the computer screen. The feedback bar presented the classification certainty as computed by the g.tec BCI classifier, which relies on the CSP+LDA approach. The classifier was calibrated for each subject based on the data of the latest run while the subject took a break between the runs.

**Fig 2:**
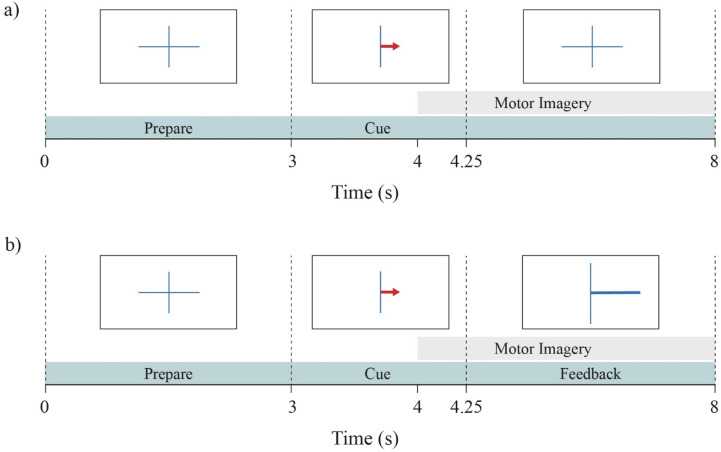
The time course of each trial in the BCI task. (a) shows the calibration run and (b) the feedback runs. In all trials, participants saw a fixation cross and thereafter an arrow pointing to either left or right, which indicated the corresponding hand for the MI task in the trial. In feedback runs, the blue bar indicated the direction and certainty of the classifier’s prediction in order to feedback to the participants. The grey area indicates the time course of the MI task.

In total, participants performed 120 MI trials. Each MI trial took eight seconds. The timeline of each trial is shown in Fig 2. It started with a fixation cross that was displayed in the center of the screen for three seconds. In the next 1.25 seconds, a red arrow cued the direction of the trial; the subject had to imagine squeezing their left hand if the arrow pointed to the left and their right hand if the arrow pointed to the right, without tensing their muscles. During the last 3.75 seconds, the calibration run showed the fixation cross again (see Fig 2A), while the feedback runs showed a blue feedback-bar indicating the direction and certainty of the classifier’s output (see Fig 2B). Participants were instructed to stay focused on the imagination of the movement even during the feedback and try to not get distracted by it. The end of the trial was marked by a blank screen. The rest time between trials varied randomly between 0.5 and 2.5 seconds.

#### 2.1.4 EEG dataset

The signals from three participants were not recorded in a satisfactory manner due to technical issues during the experiment. Hence, only 54 participants were analyzed for this study. An epoch of 4 seconds was selected from each trial. This epoch, targeting the MI period, started at second 4 of the trial (1 second after cue presentation) and ended at second 8 (5 seconds after cue presentation), which is in line with the study of [41]. The selected time segment is indicated with the grey area in Fig 2.

### 2.2 Machine learning model

The ML approach consisted of preprocessing the signals, constructing CSP filters for feature extraction and an LDA model for classification of the left vs. right classes.

CSP is a feature extraction technique that selects spatial filters from multi-channel signals and then linearly transforms EEG data into a subspace with lower dimension that maximizes the variance of one class while minimizing the variance of the other class [33, 42]. While research continues advancing other feature extraction methods [43], CSP remains the most widely used algorithm in binary MI-BCIs due to its computational simplicity and improving signal to noise ratio [44, 45]. The output of CSP can be used as input for the LDA classifier in order to distinguish the classes of MI task.

LDA is a dimensionality reduction model that works on the concept of minimizing the ratio of within-class scatter to between-class scatter while keeping the intrinsic details of the data intact [46]. Hence, LDA creates a hyperplane in the feature space based on evaluation of the training data to maximize the distance between the two classes and minimize the variance of the same class [47, 48]. LDA is very popular for binary classification of the MI task [39].

In this study, the g.tec BCI classifier, which uses CSP+LDA method, was employed to perform online classification. In addition to the real-time accuracies from this classifier that were used in this study for identifying the low and high performer groups, we reconstructed the CSP+LDA pipeline in an offline classification to provide a fair comparison between the two DL and ML approaches.

#### 2.2.1 Architecture

EEG signals recorded from the participants were pre-processed and temporally filtered to remove artifacts. Data containing bad impedance, error in recording, or excessive movement-related noise were removed (3 subjects, see 2.1.4). Then the EEG signals corresponding to the onset of MI task (second 4 to 8, see Fig 2) were selected and taken into account [49]. Thereon, Filter Bank Common Spatial Pattern (FBCSP) was used to extract subject-specific frequency band of 7–30 Hz from the data through the implementation of fifth order Butterworth [49, 50]; see mathematical details in [49].

FBCSP was used because it is instrumental in discriminating the binary classification of EEG measurements [49, 51, 52]. It should be noted that CSP is highly dependent on the selection of frequency bands, however there is no optimal solution to select the right filter bank [53]. Using a filter bank before CSP helps to improve the accuracy level of the model [54]. A wide range of 7–30 Hz is usually adopted for CSP when used for MI classification [53]. Hence, the frequency bandwidth was kept between 7–30 Hz covering the mu and beta bands that are required to analyze Event-Related Desynchronization (ERD) and Event-Related Synchronization (ERS) from the MI brain signals.

After the pre-processing and filtering steps, the 120 MI trials of each participant were concatenated and randomized. CSP algorithm was performed on each participant’s data using the ‘*scikit*’ package in Python [39]. CSP extracted the spatially distributed information from the output of FBCSP by linearly transforming the EEG measurements in order to define discriminative ERD/ERS features [49, 51, 52] according to Eq 1 [51].


$$Z_{b,i}=W_{b}^{T}E_{b,i}$$ (1)

Here, E_b,i_ represents EEG measurement of the b^th^ band-pass filter in the i^th^ trial. It has the size of c×t, where c is the number of channels and t is the number of EEG samples per channel. W_b_ is the CSP projection matrix of the size c×c and T denotes transpose operator. W_b_ is calculated by the CSP algorithm by solving the eigenvalue decomposition problem [51]. Z_b,i_ is the output of transformation (E_b,i_ after spatial filtering), which maximizes the differences in the variance of the two classes.

Once feature extraction was completed, ‘*scikit*’ package was again used to implement the LDA classifier in order to reduce the dimensionality of the sub-bands and to perform binary classification [55], see [56] for details on LDA.

### 2.3 Deep learning model

The DL model was constructed by feeding raw EEG signals directly into a CNN model. CNN is a feed-forward Artificial Neural Network (ANN) model and has a sequence of layers where every layer is the output of an activation using a differential function [35]. In a CNN, the inputs are assembled to different layers of neurons, each representing a linear combination of the inputs [57]. The learning process involves modification of the parameters by adjusting weights between different layers in order to achieve the desired output [58]. The learning continues until the training set reaches a steady state where the weights become consistent and an optimal output is reached [58]. During the training phase of the CNN model, different layers can extract features at a different level of abstraction. The initial layers learn local features from the raw input, and the end layers learn global features [34].

#### 2.3.1 Architecture

**Fig 3:**
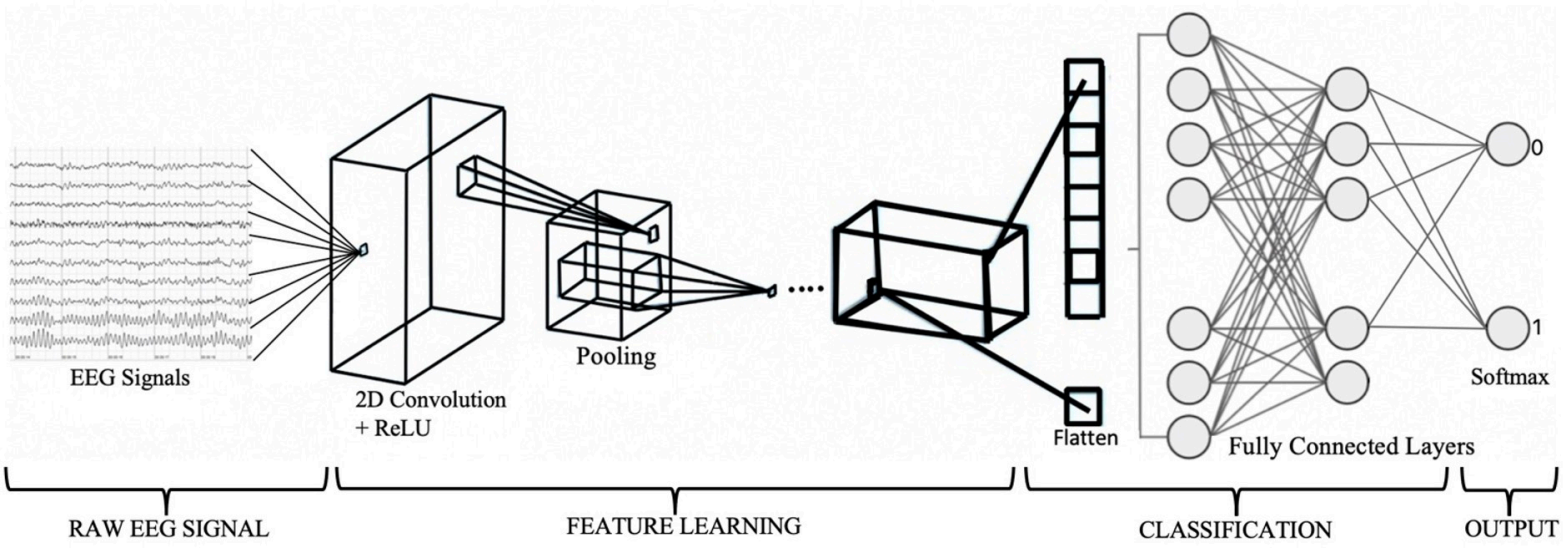
CNN architecture.

A 2D CNN model was constructed using ‘*keras*’, a high-level neural networks API written in Python [59]. Fig 3 shows the architecture of the proposed CNN model.

The first two components of the architecture are the number of convolution filters used and the kernel size that specifies the height (columns) and width (rows) of the 2D convolution window. These were set to 30 and 5×5 respectively. The dimensions of the input shape applied were 1×4×4. Convolution layers are executed according to Eq 2 [60],

s(t)=(x*w)(t) (2)

where x is the input matrix, w is the kernel and s is the resulting feature map for each time index t.

In order to compute a network’s hidden layers, activation functions should be implemented [60]. For this task, Rectified Linear Unit (ReLU) was used. ReLU conducts simple mathematical operations on input x using Eq 3, preserving characteristics that result in good generalization with less computational cost than other approaches.


R(x)=max(0,x) (3)

Moreover, ReLU has the advantage of the speed and overcoming gradient leakage issue when compared with other activation functions [57].

Max pooling was added to the model in order to downsample the input and refrain from losing important data features. The size of 2×2 was used based on the works of [61, 62]. This selected the maximum value from each 2×2 window on the feature map and used it as input for the following layer. The output of max pooling was flattened into a vector of input data by executing a flatten layer [60]. Subsequently, three dense layers were added. The first two implemented a linear function in which all inputs were connected to all outputs by a specific weight [63]. The units of these were set to 256 and 128 and were activated by ReLU functions. The final dense layer’s units were fixed to 2 as this was the number of class labels in the data. Finally, Softmax was applied to the last (output) layer as an activation function, used for class classification tasks [60], following Eq 4:

$$softmax{\left(z\right)}_{i}=\frac{e^{z_{i}}}{\sum_{k=1}^{N}e^{z_{k}^{}}}$$ (4)

where input matrix z_i_ is converted to a categorical probabilistic distribution.

#### 2.3.2 CNN model compilation

The hyperparameters implemented in the 2D CNN model’s compilation phase are the loss function, the optimizer and the evaluation metric. Since the dataset has two target labels (right and left), the loss function categorical cross-entropy was applied. The optimizer *‘Adam’* was used because it is a widely used gradient-based optimization of stochastic objective functions [64]. An essential parameter of *‘Adam’* is the learning rate, which regulates the modification of the model based on the error obtained from the updated weights [64]. For the task at hand, the learning rate was set to its default value of 0.01. The evaluation metric was set to accuracy to delineate how well the CNN model could classify left vs. right MI EEGs [60].

#### 2.3.3 CNN model fit

During model fitting, a specified batch size and number of epochs need to be adopted for backpropagation to take place [65]. The batch size greatly influences the time to converge and the amount of overfitting [66]; a big batch takes into account many samples to calculate a gradient step and therefore might slow down the model training [60]. On the other hand, small batch sizes can supervise variation in the distribution. The batch size for the 2D CNN model was set to 264.

An epoch in DL means that all the samples in the training set are traversing through the model once [65]. This helps the network to see previous data for readjusting the model parameters in order to reduce any biases. The neural network updates the weights of the neuron during each epoch [67]. However, there is not any prescribed method to calculate how many epochs are required for a particular model. [68] stated that different values of epochs should be tried until the learning curve of the model moves from underfitting to an optimum level and until overfitting attributes start showing up, then the subsequent epoch size should be deemed as the threshold for the model. Thus, as long as both training and test accuracies are increasing at an equivalent rate, the training of the model should continue [69]. Considering the arguments from [64, 69], 500 epochs per subject was deemed to be the threshold for the CNN model.

### 2.4 Evaluation

For both models, the data was split into 80% training and 20% test data [23] and the mean accuracies for all subjects in the training and test phases were calculated.

In order to compare the performance of CSP+LDA and CNN models, the difference between the two models’ accuracies *(ΔAccu = Accu*_*CNN*_*−Accu*_*CSP+LDA*_*)* was obtained for each subject. This was done to give greater validity to the findings as inter-subject variability can affect the overall performance of a classifier [70]. Next, subjects were divided into two groups of Low and High Performers by applying a median split to their online BCI accuracy (*Mdn* = 52.14%), resulting in 27 subjects in each group. The variable *ΔAccu* was then compared between the two groups.

In addition to the overall prediction accuracy, we extracted F-score metric for each class of *‘left’* or *‘right’* MI. F-score is the harmonic mean of the precision and recall metrics and demonstrates the discriminant power of the model for each existing class in the data. Previous research has shown that the BCI user handedness plays a role in lateralization of ERD/ERS during the MI task [71]. In our study, all subjects were right-handed, therefore it was expected that the errors made by the model would be more for one MI class than the other.

## 3 Results

Table 1 gives the mean training and test accuracies across 54 subjects for the CNN and CSP+LDA models. The CNN model reached an average training accuracy of 80.58% (*SD* = 5.01) and an average test accuracy of 69.42% (*SD* = 4.97), whereas the average training and test accuracies for the CSP+LDA model were 52.54% (*SD* = 5.12) and 52.56% (*SD* = 2.08), respectively.

**Table 1:** Comparison between training and test accuracies of CNN and CSP+LDA models.

Model	Training Accuracy *(N = 54)*		Test Accuracy *(N = 54)*	
	Mean	*SD*	Mean	*SD*
**CNN**	80.58	*5*.*01*	69.42	*4*.*97*
**CSP+LDA**	52.54	*5*.*12*	52.56	*2*.*08*

The obtained accuracies for both CNN and CSP+LDA models were normally distributed as evaluated with Shapiro-Wilk test (CNN: *W* = 0.98, *p* = .66; CSP+LDA: *W* = 0.97, *p* = .12). Therefore, a pairwise t-test was employed to compare the test accuracies obtained from the DL classification method to those of the ML approach (*t*(53) = 22.12, *p* < .001). This indicated that the CNN classifier significantly outperformed the CSP+LDA approach by 15.32 to 18.38% within the 95% confidence interval.

**Fig 4:**
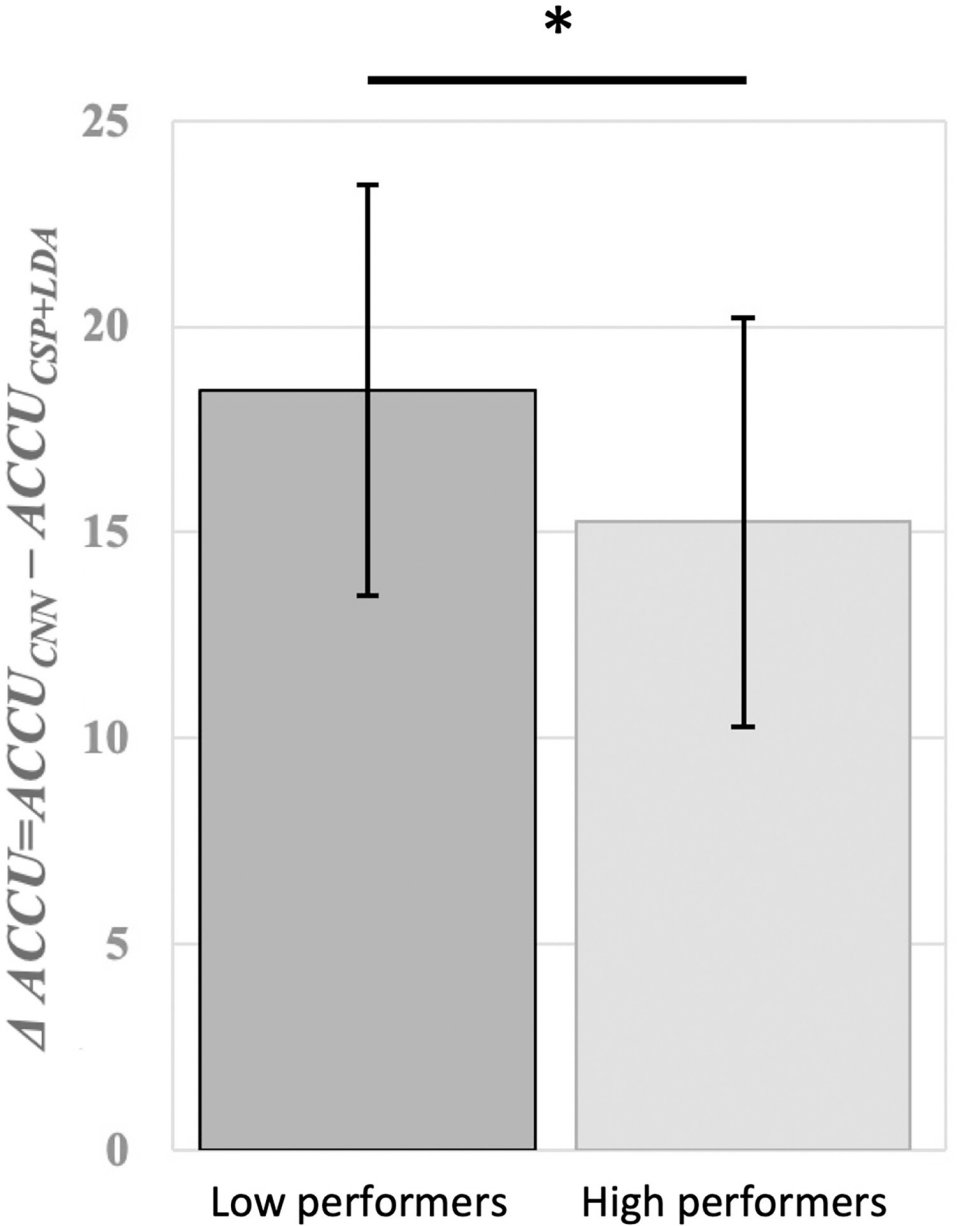
Mean difference between accuracies of CNN and CSP+LDA models (*Accu*_*CNN*_*−Accu*_*CSP+LDA*_) for Low Performer and High Performer groups. Low Performers showed significantly higher improvement in MI-BCI accuracy after using a CNN classifier.

The mean performance difference between the two models *(ΔAccu = Accu*_*CNN*_*−Accu*_*CSP+LDA*_*)* for the Low and High Performer groups is demonstrated on Fig 4. On average, the CNN model increased the accuracy rate of the Low Performers by 18.46% (*SD* = 4.98%) and the High Performers by 15.25% (*SD* = 5.81%). The obtained *ΔAccu* values for both Low Performer and High Performer groups were normally distributed as evaluated with Shapiro-Wilk test (Low Performers: *W* = 0.96, *p* = .47; High Performers: *W* = 0.98, *p* = .84). Therefore, an independent t-test was employed to compare them, revealing a significantly higher improvement of classification performance by the CNN model for Low Performers (*t*(26) = 2.18, *p* < .05).

**Fig 5:**
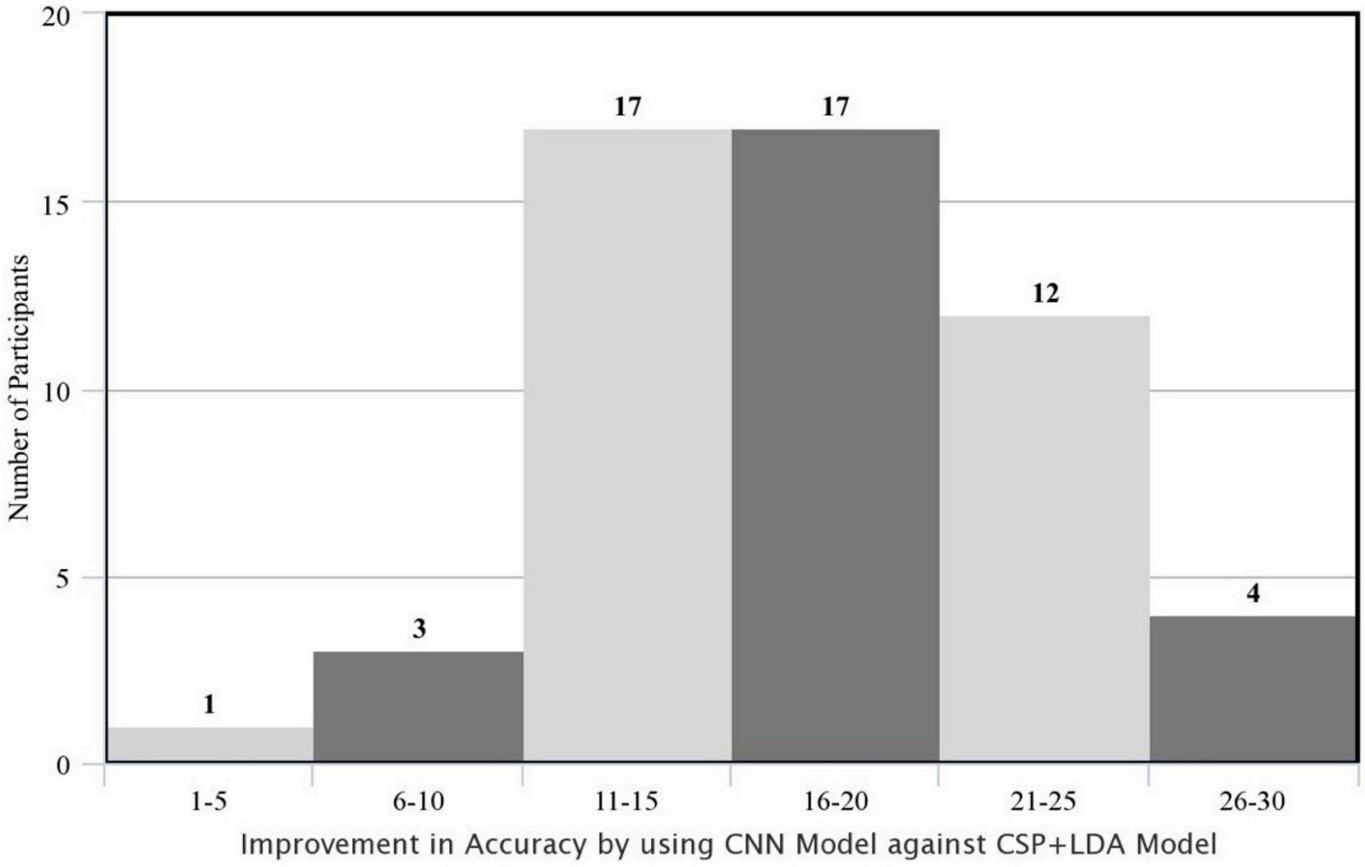
Improvement in the accuracy rate of the subjects using CNN model against CSP+LDA in percent points (i.e., absolute difference between the two accuracies; AccuCNN–AccuCSP+LDA).

Additionally, the subject-wise comparison of the models revealed that the DL approach achieved a higher accuracy level for all subjects with a minimal difference of 2.37% and maximal difference of 28.28%. Fig 5 illustrates the number of subjects for whom the CNN model showed accuracy improvement in 6 bins of 1–5%, 6–10%, 11–15%, 16–20%, 21–25% and 26–30%. From this figure, it can be inferred that the CNN model outperformed the CSP+LDA model by more than 11% accuracy for 92.59% of the participants. Therefore, it can be concluded that CNN was able to extract intrinsic features from the EEG signals and thereon, performed classification with higher accuracy level.

Finally, F-Score was calculated for each class in order to measure the predictive power of the classifiers with respect to the ‘*left*’ and ‘*right*’ MI movements. Table 2 summarizes the mean and SD of F-Scores across all subjects obtained by the CNN and CSP+LDA models in regard to each MI class. As can be seen in this table, the CNN model achieved higher F-Score values for both ‘*left*’ and ‘*right*’ hand prediction compared to the CSP+LDA model. A pairwise t-test comparing the F-Scores of the two models found a significant difference for both ‘*left*’ MI movements (*t*(53) = 18.28, *p* < .05) as well as ‘*right*’ MI movements (*t*(53) = 19.47, *p* < .05) favoring CNN as a classifier beyond CSP+LDA approach.

**Table 2:** Average F-score obtained by the CNN and CSP+LDA models for each MI class.

Evaluation Metric	CNN Model				CSP + LDA Model			
	Left Hand *(N = 54)*		Right Hand *(N = 54)*		Left Hand *(N = 54)*		Right Hand *(N = 54)*	
	Mean	*SD*	Mean	*SD*	Mean	*SD*	Mean	*SD*
**F-Score (%)**	69.07	*5*.*35*	68.59	*5*.*23*	52.93	*3*.*67*	51.83	*3*.*56*

## 4 Discussion

In order for a BCI system to operate optimally for all users, it is crucial to devise a classification model that can learn from each individual’s brain signals and recognize task-related patterns with high accuracy. The main goal of this study was to validate whether a deep learning approach employing raw EEG signals could outperform the traditional machine learning based MI-BCIs, particularly for inefficient users. Two models; CNN (DL approach) and CSP+LDA (ML approach) were trained and tested using a large EEG dataset from 54 subjects who conducted MI task during a BCI interaction. Results showed that the CNN model produced significantly higher classification accuracy for the MI task as compared to the CSP+LDA model for all users, but especially benefitted low aptitude users by increasing their BCI performance significantly more than high aptitude users.

BCI inefficiency has long been seen as a human factor problem in the literature. Only recently, [28] suggested that DL approaches might increase accuracies for low aptitude performers, thereby enabling some of them to reach performance above the threshold of 70% accuracy. This study confirmed their finding by providing statistically validated results supporting the superiority of the CNN model in capturing intrinsic oscillation patterns associated with the MI task in inefficient users. That is, CNN significantly improved the classification accuracy for those users whose performance was lower when the conventional CSP+LDA model was adopted. This supports the arguments by [13], who criticized the notion of ‘BCI illiteracy’ and stated that poor BCI performance should not be always blamed on the user. Deducing from this and also from previous studies [23–26, 28, 30–32], it can be concluded that regardless of the users’ ability to generate MI-specific sensorimotor oscillations, DL models are effective in extracting intrinsic features from EEG signals and therefore, can perform MI classification with higher accuracy level.

Until now, an in-depth analysis of the predictive power of DL classifiers for different BCI user groups was still missing. Past studies on DL approaches often employed limited number of subjects, which did not represent the large inter-subject variability that exist among users [72]. For instance, [25, 26] employed a dataset with only 9 subjects and [24] trained their CNN model with data from only 5 subjects. However, with recent release of larger scale EEG datasets [e.g., 73, 74], there have been more attempts on employing DL models on signals from large number of participants [e.g., 28, 30–32], confirming the relevance and timeliness of this study in the BCI field. Although these studies report the same conclusion for superiority of the DL approach in MI-BCI classification, their methodology and approach in building the DL model is different from our study and none of them had attempted the comparison of the model performance between different user groups. For instance, [28] trained a CNN model with high density EEG (64 channel) to classify a 4-class MI task, [31, 32] focused on feature representations in the model and [30] attempted transfer learning with the CNN model for subject-independent classification. Our study dissociates itself from these prior studies by applying a simple CNN architecture for end-to-end learning and providing evidence for suitability of the DL approach for inefficient BCI users.

The accuracy level achieved by the DL model in this study might initially seem insufficient when compared to [24, 26], however, this difference can be explained by various pre-processing and feature engineering techniques that were employed by these two studies. Unlike past research, this study focused on evaluating the performance of CNN model without implementing any fine-tuning techniques and by directly feeding raw data into the model. The motive for this approach was to show the efficacy of deep learning models in exploiting information from raw data without any need for feature extraction. This makes deep learning models computationally more effective by eliminating the costly steps of pre-processing and feature extraction. Additionally, such neural networks can handle noise in EEG signals better than ML models and thus can provide a more robust performance in real-time BCI applications [29]. Other deep learning methodologies such as recurrent neural nets or autoencoders have also been explored in previous studies, however CNN remains the most prevalent and consistently showed better performance than other approaches [75]. Of course, these models can be made increasingly complex by adding layers. Future research could pinpoint the ideal model construction for MI-BCI classification.

The low performance obtained in the ML approach has to be compared to the online classification accuracies presented in [15], where the average classification accuracy was 74.17%. This could be explained by different architectures: The online classification of [15] was conducted by g.BSanalyze software (g.tec Medical Engineering, Austria). In this model, baseline non-feedback data is provided to the model to calibrate the classifier for each subject before the actual classification runs. In addition, the lack of removal of bad trials in our ML approach may explain a difference in the acquired classification accuracies. Also, in [15] subjects were trained upon online classification, optimizing performance for that specific processing pipeline. Therefore, to make a fair comparison with our DL model, we employed a ML approach using offline classification with no prior training and calibration of the system.

The BCI performance is a product of the interplay between the BCI system and the user [76]; therefore, the importance of user training and the ‘*human in the loop*’ cannot be overlooked. To further increase the performance of inefficient users, factors that relate to the user might be investigated too: Motivation and feedback play an important role in user’s learning of the MI task [1, 77]. Hence, interaction with a MI-BCI could be improved by inducing more engagement during the task [77], giving more detailed instructions on cognitive strategy and reducing the cognitive load during BCI training [77]. Past studies have shown that embodied feedback in form of virtual or robotic hands can also improve interaction between the user and the BCI system [78, 79]. Future studies should attempt to replicate the results of this study with a more engaging and realistic feedback that could lead to generation of more distinguished brain patterns by the user at the data collection stage as greater differences in activation patterns between high and low performers might be observed.

Yet, another challenge in the development and application of MI-BCI systems is their long calibration time [80]. Since offline classification is not suitable for continuous BCI control [81]—fluidly controlling an external device is not equal to outputting one command at the end of a trial [82]—Stieger et al. [28] simulated continuous control by providing feedback based on the estimated output from the CNN model every 40 milliseconds. They showed that CNN applied on all 64 electrodes made decisions earlier and could therefore be applied to make faster predictions in continuous control compared to CNN trained on only motor area electrodes. Their proposal suggests that CNN is applicable for continuous control and thereby provides the opportunity for future research on how such continuous feedback might benefit inefficient users during BCI training. Other attempts to reduce the calibration time or completely eliminate it, have proposed transfer learning in which common information across subjects or sessions is mined and used for training of the classifier to improve the prediction for a new target subject [83]. Most transfer learning methodologies focus on extracting features and adapting them from the source subject(s) to the target subject, whereas in DL models with an end-to-end decoding, the neural network itself should be able to do this with little data pre-processing [30]. Future research could compare the performances and required calibration time between continuous CNN and transfer learning in order to devise a better training for inefficient users.

In sum, this study aimed to show the potential of DL for MI-EEG classification as opposed to the state-of-the-art ML classifiers particularly for inefficient users. Our results showed that compared to the conventional CSP+LDA model, the CNN model, which was trained and tested on raw EEG signals, could achieve significantly higher classification performance for all users, but especially for inefficient users. The novelty of this research lies in employing a large group of BCI users, which allowed comparison of user groups (high vs. low performers), employing raw EEG signals for training of DL model and investigating how different classification approaches contribute to user performance. Applying DL to BCI applications is a burgeoning field, which requires further dedicated effort for development and validation. The applications of this finding might be elaborated to other BCI applications aiming at cognitive load [84] or attention [85]. This study presents promising findings for the design of future BCI applications that are robust to individual differences among users with respect to the MI task. Future studies should deploy the proposed CNN model on new subjects to evaluate the real-time performance of the model and to examine whether the same model can be employed for subject-independent classifiers.

## 5 Conclusion

In this research, we evaluated the benefits of DL in improving the performance of motor imagery BCIs for different user groups. We extracted the performance of a CNN model trained on raw EEG signals from 54 subjects and statistically compared it to that of CSP+LDA, which is a popular ML classifier for binary classification of the MI task. The results revealed that the CNN model significantly outperformed the traditional ML pipeline by increasing classification accuracy for all subjects, but especially the inefficient BCI users whose performance improvement was significantly larger than high performers. We conclude that DL classifiers show promise for future MI-BCI applications as opposed to the current state-of-art ML-based BCI systems, which demand extensive effort in pre-processing and feature extraction and yet are impractical for some users. Future studies should further investigate the robustness of the proposed CNN model in real-time MI-BCI applications and their effectiveness in establishing a better interaction between inefficient users and the BCI system.
